# Professionalism Program Evaluation and Its Impact on Undergraduate Medical Students

**DOI:** 10.7759/cureus.53051

**Published:** 2024-01-27

**Authors:** Sarah Amin, Sundus Ambreen, Noor Ul-Ain, Tasneem Murad, Khadeejah Sajwani, Aasma Qaiser

**Affiliations:** 1 Medical Education, National University of Sciences and Technology (NUST) School of Health Sciences, Islamabad, PAK; 2 Forensic Medicine, Islamic International Medical College Trust, Rawalpindi, PAK; 3 Medical Education, Islamic International Medical College Trust, Rawalpindi, PAK; 4 Anatomy, National University of Sciences and Technology (NUST) School of Health Sciences, Islamabad, PAK; 5 Medical Education, CMH (Combined Military Hospital) Kharian Medical College, Kharian, PAK

**Keywords:** impact, undergraduate medical students, medical education, program evaluation, qualitative study, professionalism

## Abstract

Objective

The study aims to evaluate the effectiveness of a professionalism program by conducting focus group discussions (FGDs). The research focuses on understanding how the program influences the development of professionalism skills among medical students, as well as their perceptions and experiences regarding the program. The study's objectives revolve around identifying the strengths and weaknesses of the program from the perspective of the students.

Methods

This was a qualitative study done after obtaining approval from the Institutional Review Committee, Islamic International Medical College. Students of 4th year Bachelor of Medicine and Bachelor of Surgery (MBBS; 56 students) were selected for the study. A convenience sampling technique was used to select the participants for FGDs. Seven separate FGDs were conducted, with eight participants in each group. All the FGDs were audiotaped. Data were transcribed and translated. Data were analyzed using the thematic analysis on Atlas. Both obvious and hidden content were analyzed. Codes and themes were identified, which were then finalized with consensus. Codes were then categorized into sub-themes, and finally, themes were generated.

Results

Participants identified the problems associated with applying professionalism during FGDs. These challenges encompassed hefty workloads and a perceived disparity between theoretical knowledge and practical implementation. The students highlighted the importance of practical teaching methods, the cultivation of role models, the alignment of curriculum with real-world experiences, and the revision of assessment methods. This study analyzes the obstacles and potential advantages associated with professionalism education and presents significant perspectives on improving it for upcoming healthcare practitioners.

Conclusion

Professionalism is a crucial component, and each graduate of a medical school should be fully qualified as a professional after graduation. As we want our future doctors to be skilled at both professional qualities and diagnosis, it is crucial that medical institutions teach professionalism.

## Introduction

Professionalism is defined as the manner by which individual doctors fulfill the medical profession's relationship with society [1]. Several organizations involved in physician education and training, including the American Board of Internal Medicine, the European Federation of Internal Medicine, the Accreditation Council for Graduate Medical Education, and the Association of American Medical Colleges, have identified the essential elements of professionalism that each physician must possess. Professionalism, while based on technical competence, also addresses humanistic issues such as caring and compassion. There is no commonly acknowledged list of professional behaviors that may be used to guide instruction or correct assessment. Most international literature agrees on six major characteristics: altruism, accountability, excellence, duty, honor and integrity, and respect for others [2].

Professionalism assessment and students' professional behavior have become an increasingly significant element of the medical education curriculum [3]. Promoting professional behavior is currently a top priority across the medical education continuum. Assessment is a vital component of this program.

Recently, Byszewski et al. [4] conducted a survey of students and teachers at the University of Ottawa to acquire insight into the existing professionalism curriculum. According to their findings, students regarded role modeling as the most effective teaching method, and present professional assessments required modification.

According to a recent study, the Pakistani medical community is also working to rectify professional shortcomings through conservation, research, teaching, and evaluation [5]. Our study adds to our understanding of the student perspective on professionalism by evaluating the professionalism program and its impact on undergraduate medical school and makes recommendations for improving the professionalism curriculum at Islamic International Medical College (IIMC).

An increasing body of research suggests that this goal is entirely attainable [6]. The purpose of assessing professionalism is to identify whether or not trainees have learned this basic ability and what type of shortcomings they need to rectify [7]. Furthermore, unless we hold students accountable for displaying these characteristics in high-stakes assessments, they are unlikely to prioritize meeting the ostensible criteria. Stern stated clearly, "If it can't be measured, it can't be improved," and "they don't respect what you expect; they respect what you inspect" [8].

Thus, efforts to improve students' professional performance should begin with the formulation and execution of credible assessments of the characteristics that are desired.

## Materials and methods

This was a qualitative study done after obtaining approval from the Institutional Review Committee of Islamic International Medical College (Ref #: Riphah/IIMC/IRC/23/3051). The college is located in the heart of Rawalpindi, the curriculum is integrated, and the college places emphasis on Islamic values as well. Students undergo a professionalism module as a part of their integrated curriculum and it is completed in the 4th year. Students of 4th year Bachelor of Medicine and Bachelor of Surgery (MBBS; 56 students) were selected for study and initial years were not included in the study because of their still ongoing professionalism module. A convenience sampling technique was used to select the participants for focus group discussions (FGDs). Seven separate FGDs were conducted with eight participants each, two FGD groups were of male students and five FGD groups were of female students, informed consent was taken and participants were also briefed about the study objectives. A medical educator and a faculty member moderated the FGDs. The FGD guides were used after pilot testing. All the FGDs were audiotaped. The Urdu language was also used in addition to English for communication in these FGDs, as participants could express their opinions & experiences better in Urdu. Data were transcribed and translated into English as well; all personal information was removed to maintain confidentiality. Researchers discussed the transcript amongst themselves to ensure credibility. Data were analyzed using thematic analysis. Both obvious and hidden content were analyzed. Codes and themes were identified, which were then finalized with consensus. Codes were then categorized into sub-themes, and finally, themes were generated.

A manual thematic analysis of the data was done. In the first step, there was familiarization with the data, and then the creation of the initial code list manually. Data were coded, and the code list was revised. In the second step, codes were grouped, categories were generated, and themes were generated. In the third and last step, there was confirmation of themes and an explanation of themes in the results. The researchers went through audio files twice and data were again reviewed by another observer and important points were compared with the one already noted for data triangulation.

Questions asked in FGDs are presented in Table 1.

**Table 1:** Questions asked in focus group discussions

Questions	Attributes
Do you think the professionalism module helped in building up the excellence concept in you?	Excellence
Did this module of professionalism teach you how to be more respectful toward patients and people in general?	Respectfulness
How did your dutifulness toward your profession improve after this module?	Dutifulness
Did this module help you in building up your moral values and are you being honest with your work after going through this module?	Integrity
Did your sense of responsibility improve after going through the module?	Accountability
Did this module help you in establishing teamwork qualities?	Teamwork
Was the module of professionalism in accordance with the Islamic values taught at Islamic International Medical College?	Islamic values

These questions were sent to three senior medical educationists across Pakistan for validity purposes. Pilot testing was also done and it took around 25-30 minutes for one FGD session.

## Results

After going through the FGDs and sorting out the results, many sub-themes and respective themes were identified. These major identified themes are shown in Table 2.

**Table 2:** Results of focus group discussions

Subthemes	Themes
Theoretical learning challenges in practical application and the need for practical approaches to teaching and learning	Learning professionalism theory vs. application of knowledge
Core attributes of professionalism and integration of attributes into the medical curriculum	Attributes of professionalism
Importance of role modeling of professionalism and perceived lack of professionalism among seniors	Deficit role modeling
Workload and time constraints limited patient interaction and the gap between theory and practice	Challenges in implementing professionalism
Need for practical teaching, need for role models, alignment of theory and practice in teaching professionalism, and revision of assessment methods of professionalism module	Suggested improvements in the professionalism module

Students highlighted many important aspects regarding the professionalism module and recommended multiple changes to improve the efficacy of the module and practicality.

Theme 1: Learning professionalism theory vs. application of knowledge

The participants articulate that although they have acquired knowledge regarding professionalism in a theoretical context, they encounter difficulties when attempting to implement these concepts in real-world environments, such as hospitals. The participants held the belief that theoretical instruction did not sufficiently equip them for practical scenarios. The participants articulate challenges encountered while attempting to implement the tenets of professionalism within practical contexts, with a specific emphasis on the hospital environment.

There is a consensus among the participants on the inadequacy of academic learning in isolation, emphasizing the significance of incorporating practical and experiential methods to cultivate professionalism.

"We theoretically read confidentiality, excellence & integrity, but practically we didn’t apply anything yet." P1FGD2

"We are being taught in lectures and SGDs, but these are useless unless we are taught these things practically at the workplace." P5FGD2

“It is impossible to truly understand professionalism through lectures and textbooks; instead, one must experience and practice it in healthcare settings.” P2FGD4

"Lectures are informative, but they don't prepare us for the practical realities of professionalism in healthcare."

Theme 2: Attributes of professionalism

The concept of professionalism encompasses a set of fundamental traits that are widely regarded as necessary. These attributes include confidentiality, excellence, integrity, accountability, altruism, respect, and punctuality. The participants appreciated the incorporation of these traits into the curriculum for medical students to become future healthcare providers. They were satisfied with including these attributes as part of the module. Professionalism requires confidentiality; we must protect patient information and respect their privacy.

Excellence in our work preserves our professionalism. We must strive for the utmost level of care. Integrity and ethical behavior are non-negotiable professional characteristics. In our employment, we must uphold the highest moral standards.

"Being responsible for our actions is an indication of our professionalism. We must act with honesty and accept responsibility for our choices." P4FGD4

Professionalism is founded upon altruism. We must always prioritize our patients' well-being and place their needs first. Professionalism entails delivering patient-centered care and prioritizing the patient's comfort and happiness.

"Interactions based on courtesy are a fundamental aspect of professionalism. We must treat patients and coworkers with compassion and courtesy." P2FGD3

"As prospective healthcare providers, we must incorporate these professional characteristics into our daily healthcare practice. It is about translating these principles into actionable steps to benefit our patients and colleagues." P5FGD4

Theme 3: Deficit-related role models

The participants underscored the necessity of having role models that exemplify professionalism through their actions in their professional environment. Numerous participants believed their senior physicians and professors were not good role models of professionalism, detracting them from their educational experience.

Many participants emphasized the significance of having role models in the professional environment to exemplify professionalism.

"It is invaluable to have role models who demonstrate professionalism through their actions. They motivate us to pursue excellence in our own work." P3FGD1

"Unfortunately, some of our senior physicians and professors show a lack of professional attributes, which has a negative impact on our educational experience. We require improved or more in-practice role models." P1FGD3

"When senior physicians lack professionalism, it sends students the incorrect message. We seek mentors who exemplify the values we are acquiring." P2FGD3

"The importance of role models in shaping our professionalism cannot be overstated. Their deeds speak louder than words, and we look to them for direction." P2FGD5

Theme 4: Challenges in applying professionalism

Participants cited several challenges in implementing professionalism, including heavy hospital workloads, limited patient interaction, and a lack of practical teaching. The disconnect between what is taught in lectures and what is observed in the hospital setting is a significant challenge.

Participants also quoted several challenges in implementing professionalism, including hefty hospital workloads, limited patient interaction, and a lack of practical teaching. A significant obstacle is the disparity between what is taught in lectures and what is observed in hospitals. This encompasses the obstacles associated with the substantial workloads experienced in hospital settings and the constraints imposed by restricted time for cultivating and demonstrating professionalism.

"Limited patient interaction refers to a situation with a restricted level of engagement between healthcare providers and patients. This study centers on the difficulties that arise because of the restricted opportunities for engaging with patients, which consequently impede the practical implementation of professionalism." P1FGD5

The discrepancy between the content taught in academic lectures and the practical experiences encountered in medical settings is a substantial challenge in cultivating and applying professionalism. A hospital's heavy burden can make it difficult to maintain professionalism. Occasionally, doctors are simply attempting to keep up with the pace.

"We do not always have the opportunity to apply what we've learned due to limited patient contact. This is a challenge we must overcome to become better professionals." P2FGD5

The disparity between what we learn in lectures and what we observe in hospitals can be discouraging. It is difficult to reconcile the two. The burden of work is actual, but this should not prevent us from maintaining professionalism. We require strategies for overcoming these obstacles.

Theme 5: Suggested improvements by students

The necessity of practical teaching is underscored by participants who highlighted the significance of integrating practical teaching approaches within professional education.

An individual quoted the following: "Relying solely on theoretical knowledge does not provide them with the necessary abilities and mentality to effectively engage in professional practices." P3FGD4

Pragmatic pedagogical approaches encompass the utilization of authentic situations, simulations, and experiential learning opportunities, enabling learners to effectively apply acquired knowledge concretely.

The formation of role models within the healthcare profession who embody professionalism is emphasized by the participants.

"Individuals acknowledge the significant impact that role models have on molding their own behavior and beliefs." P1FGD5

The presence of mentors and experienced professionals who continuously exhibit professionalism has the potential to serve as a source of inspiration and guidance for the upcoming cohort of healthcare practitioners.

"The convergence of theory and practice in the field of study pertains to the aspiration for enhanced congruity between the content conveyed to us during theoretical lectures and the practical encounters we have within actual hospital environments." P2FGD5

The participants articulate a discrepancy between the conceptualized ideals of professionalism that are conveyed in lectures and the pragmatic obstacles they face in real-world scenarios. It is advocated "that endeavors should be undertaken to ensure the smooth applicability of academic knowledge to practical situations in our daily professional activities." P5FGD1

The participants proposed a revision of assessment methodologies to enhance the accuracy of evaluating professionalism and its practical implementation.

"The existing methods of assessment may not adequately capture the intricacies of professionalism and its practical application." P5FGD5

Through the implementation of revised evaluation methods, the aim is to obtain more substantial feedback pertaining to their proficiency in professionalism and their overall advancement.

In general, there is more emphasis on the active learning of participants in the professionalism module. The individuals acknowledge the significance of experiential education, the influence of role modeling, the necessity for enhanced congruity between theoretical concepts and real-world application, and the possibility of enhanced assessment tools. The aforementioned recommendations are intended to augment the module's quality of education, resulting in healthcare providers who are better equipped and more proficient in their roles.

"To genuinely comprehend and apply professionalism, we need more hands-on learning experiences." P3FGD3

"Theory alone is insufficient. There should be efficient mentoring programs to bridge the gap and more role models are needed to display and teach professionalism." P1FGD5

Curriculum alignment with the content and method of evaluation is also of utmost importance and evaluation methods should reflect the essence of professionalism.

The proposed changes seek to make professionalism education more applicable and efficient, ensuring that what is learned is applied.

## Discussion

A commitment to upholding one's professional obligations, adherence to moral standards, and consideration for a diverse patient group are all examples of professionalism. In addition to demonstrating respect, compassion, and honesty, professionals are also expected to be accountable to patients, society, and the profession. They should also be dedicated to excellence and ongoing professional growth. It includes showing a dedication to the moral standards that govern the giving or withholding of clinical care, the privacy of patient data, informed consent, and commercial conduct, and also showing sensitivity and response to the age, gender, culture, and disabilities of the patients [9].

It is the utmost duty of medical institutions to produce top-class professionals. This will improve the overall atmosphere of the institution, hospitals, and society. That is the reason the professional module holds a very superior position in all the modules.

One of the most important ways of teaching professionalism to undergraduate students is role modeling. Many difficulties come up with teaching professionalism concepts. It could be intimidating to just give kids a list of what professionalism entails. Additionally, students must deal with unfavorable media role models. The doctor characters in television shows like *House* and *M.D.* frequently set an unprofessional example [10].

When FGDs were conducted with students of 4th year MBBS, many shortcomings were identified. Students had major concerns regarding the teaching strategies. The whole class session should be interactive, with a balance of contributions from teachers and pupils. It should serve and not detract from the objective. In most cases, we will need to use a combination of instructional strategies [11]. There should be more role-playing so that students can master their professional values practically.

The content of the module needs to be organized again since there is ambiguity between ethical values and professional values. Content should be more student-friendly with applicability so that we can see a major change in students once they pass out from this institution.

The medical profession's agreement with society includes professionalism, which is crucial. Based on the evidence in the literature, we must not only make wise judgments for our patients, but we must also apply those decisions in a way that is professional and ultimately beneficial to our patients. There is a correlation between specific early behaviors in medical school and unprofessional behavior later in a physician's career. These are the kinds of behaviors that need to be watched out for, and we need to make our students and trainees aware of why we are so concerned about them. With education and practice, doctors' professionalism is likely to increase.

## Conclusions

Professionalism is a very important aspect and every graduate from a medical institution should be thorough and professional once he graduates. Teaching professionalism in medical colleges is of utmost importance since we want our future doctors to be good at diagnosing as well as good at professional attributes. The professionalism module should cover all the important aspects required by doctors in their professional lives. These attributes are very important in the professional lives of medical practitioners. If we can train our medical students perfectly in their undergraduate time then we can produce better doctors with good professional attributes. Evaluation is another important aspect and must be carried out regularly. Evaluation of the module brought forward a few barriers that students were facing and suggestions have been notified to higher authorities to make some amendments.

The limitation of the study was that it was contextualized, and findings are certainly limited and do not aim for generalization. The study was conducted in one private college and there was no comparison available. In the future, studies should be conducted in more than one institution for more generalization.
